# Osmium Recovery as Membrane Nanomaterials through 10–Undecenoic Acid Reduction Method

**DOI:** 10.3390/membranes12010051

**Published:** 2021-12-30

**Authors:** Paul Constantin Albu, Andreea Ferencz (Dinu), Hussam Nadum Abdalraheem Al-Ani, Szidonia-Katalin Tanczos, Ovidiu Oprea, Vlad-Alexandru Grosu, Gheorghe Nechifor, Simona Gabriela Bungău, Alexandra Raluca Grosu, Alexandru Goran, Aurelia Cristina Nechifor

**Affiliations:** 1 Radioisotopes and Radiation Metrology Department (DRMR), IFIN Horia Hulubei, 023465 Măgurele, Romania; paulalbu@gmail.com (P.C.A.); aureliacristinanechifor@gmail.com (A.C.N.); 2 Analytical Chemistry and Environmental Engineering Department, University Politehnica of Bucharest, 011061 Bucharest, Romania; andra_d24@yahoo.com (A.F.); hussamalani32@gmail.com (H.N.A.A.-A.); ghnechifor@gmail.com (G.N.); alexandra.raluca.miron@gmail.com (A.R.G.); alexandru@santego.ro (A.G.); 3 Chemical Industries Department, Institute of Technology, Middle Technical University, Al Zafaraniyah, Baghdad 10074, Iraq; 4 Department of Bioengineering, University Sapientia of Miercurea-Ciuc, 500104 Miercurea-Ciuc, Romania; 5 Department of Inorganic Chemistry, Physical Chemistry and Electrochemistry, University Politehnica of Bucharest, 011061 Bucharest, Romania; ovidiu.oprea@upb.ro; 6 Department of Electronic Technology and Reliability, Faculty of Electronics, Telecommunications and Information Technology, University Politehnica of Bucharest, 061071 Bucharest, Romania; 7 Department of Pharmacy, Faculty of Medicine and Pharmacy, University of Oradea, 410028 Oradea, Romania; sbungau@uoradea.ro

**Keywords:** osmium nanoparticle, osmium nanoparticle–polymer membranes, 10–undecenoic acid, composite membranes, catalytic redox processes, membrane contactor, 10–undecylenic acid

## Abstract

The recovery of osmium from residual osmium tetroxide (OsO_4_) is a necessity imposed by its high toxicity, but also by the technical-economic value of metallic osmium. An elegant and extremely useful method is the recovery of osmium as a membrane catalytic material, in the form of nanoparticles obtained on a polymeric support. The subject of the present study is the realization of a composite membrane in which the polymeric matrix is the polypropylene hollow fiber, and the active component consists of the osmium nanoparticles obtained by reducing an alcoholic solution of osmium tetroxides directly on the polymeric support. The method of reducing osmium tetroxide on the polymeric support is based on the use of 10-undecenoic acid (10–undecylenic acid) (UDA) as a reducing agent. The osmium tetroxide was solubilized in *t*–butanol and the reducing agent, 10–undecenoic acid (UDA), in *i*–propanol, *t*–butanol or *n*–decanol solution. The membranes containing osmium nanoparticles (Os–NP) were characterized morphologically by the following: scanning electron microscopy (SEM), high-resolution SEM (HR–SEM), structurally: energy-dispersive spectroscopy analysis (EDAX), Fourier transform infrared (FTIR) spectroscopy. In terms of process performance, thermal gravimetric analysis was performed by differential scanning calorimetry (TGA, DSC) and in a redox reaction of an organic marker, *p*–nitrophenol (PNP) to *p*–aminophenol (PAP). The catalytic reduction reaction with sodium tetraborate solution of PNP to PAP yielded a constant catalytic rate between 2.04 × 10^−4^ mmol s^−1^ and 8.05 × 10^−4^ mmol s^−1^.

## 1. Introduction

Composite membranes made of metal or oxide nanoparticles on/in polymeric supports have been developed to improve both physical-chemical characteristics (mechanical, thermal or chemical resistance) and process characteristics (flow, selectivity, cleaning possibilities or avoidance of biodegradation) [1,2]. The nanoparticles’ characteristics (nature, morphology, dimensions) and their distributions are decisive for membrane processes’ performance [3,4,5]. A wide variety of materials, especially nanometers, have been used to prepare the composite membranes: graphene [6], fullerenes [7], carbon nanotubes [8], oxide materials, metals and their compounds [9,10,11]. Metal nanoparticles are increasingly used in the manufacture of composite membranes and related membrane processes because they provide the membranes with the ability to react selectivity, or with a biocidal effect. They include the following: silver, gold, copper, nickel, palladium, and platinum [12,13,14]. Recently, osmium nanoparticles have been used for oxidation or reduction processes with oxygen and hydrogen gas in contactor-type membrane systems [15,16].

Osmium, as osmium tetroxide (OsO_4_), is a contrast material from the perspective of studies in electron microscopy [17], but both it and its compounds ensure the following qualities of catalytic reaction processes: versatility (reduction and oxidation), selectivity, and regional and stereo-specificity [18,19,20].

The most important raw material from which osmium can be obtained is osmium tetroxide (OsO_4_), which appears as a residual compound in obtaining various metals (especially copper) by heat treatments [21]. Significant amounts of osmium are also found as waste in the laboratories of electron microscopy, microbiology, organic chemistry or biochemistry, in the form of solutions of osmium tetroxide [22,23,24]. The recovery of osmium from residual osmium tetroxide is a necessity imposed by its high toxicity, but also by the technical-economic value of metallic osmium [25,26].

The osmium recovery process involves fixing or removing oxygen from osmium tetroxide (Equation (1)):

OsO_4_ + Red → Os + Ox (1)

A wide range of reducing substances, especially organic compounds, are used to generate metallic osmium, according to Equation (1), but molecular hydrogen reduction is the most often used because the metal is obtained without impurities (Equation (2)):

OsO_4_ + 4 H_2_ → Os + 4 H_2_O (2)

However, obtaining osmium as nanoparticles according to the reaction (1) with some organic compounds may be considered when the reaction products are beneficial for subsequent applications.

An extremely important aspect of the previous research was the adhesion of osmium nanoparticles to the polymeric support on which they were obtained [15,16].

The reduction with hydrogen gas leads to aggregates of osmium nanoparticles, of micron size, on the polymeric membrane, which are disaggregated after a catalytic process, both in reduction and oxidation, creating the possibility to disperse in the reaction medium [16]. The appearance of the composite osmium nanoparticles–polymer membrane is compact, especially after the oxidation of 10–undecenoic acid (UDA) [15].

Thus, one very interesting compound, with a presumptive reducing character, is 10–undecenoic acid (or 10–undecylenic acid—UDA), which has been recently oxidized in a process catalyzed by osmium nanoparticles [15,16]. This acid is known for its multiple technical uses: obtaining polymers (polyamides, polyesters, polyurethanes), biodiesel, biomedicine, sports medicine, and cosmetics [27,28,29,30]. It is also known for chemical applications involving its bifunctional nature (alkene and carboxylic): coating of nanoparticles, spacer for stationary chromatographic phases, formation of useful compounds from functional groups (alcohols, amines, glycols, acid chloride, esters, amides) [30,31,32,33,34,35].

The subject of the present study is the production of a composite membrane in which the polymeric matrix is the polypropylene hollow fiber, and the active component consists of the osmium nanoparticles obtained by reducing an alcoholic solution of osmium tetroxide directly on the polymeric support by the reduction reaction with 10–undecenoic acid. The obtained composite membrane was morphologically and structurally characterized, and the process performances were verified in the reduction reaction of *p*–nitrophenol to *p*–aminophenol, which is important both for reducing the toxicity of the reactant and especially for the technical-economic value of the reaction product.

## 2. Materials and Methods

### 2.1. Materials

Hydrochloric acid (37%), sodium borohydride (NaBH_4_, 37.83 g/mol, pH value of 11 (10 g/L solution in water)), *i*–propanol, *t*–butanol and *n*–decanol were used without additional purification as supplied by the manufacturer (Merck KGaA, Darmstadt, Germany).

Osmium tetroxide (OsO_4_), undecylenyl alcohol (11–hydroxy–1–undecene or 10–undecen–1–ol), 10–undecylenic acid or 10–undecenoic acid (>95%), and *p*–nitrophenol, are from Sigma-Aldrich (Merck KGaA, Darmstadt, Germany).

The pure water used for the preparation of all the solutions used in the experiments was obtained with a standard system MilliQ^®^ Direct 8 RO Water Purification System (Merck, Darmstadt, Germany). The characteristic conductivity of the water obtained through the reverse osmosis system is 18.2 μs/cm.

As a membrane support material, a bundle of polypropylene fibers (surface 1 m^2^/bundle) was purchased from GOST Ltd. (GOST Ltd., Perugia, Italy). To maintain the fibers’ characteristics, they were conditioned with polyols. Their morphological, structural and process characteristics were previously presented in detail [36,37,38].

### 2.2. Procedures

#### 2.2.1. Obtaining Composite Membranes

**Figure 1:**
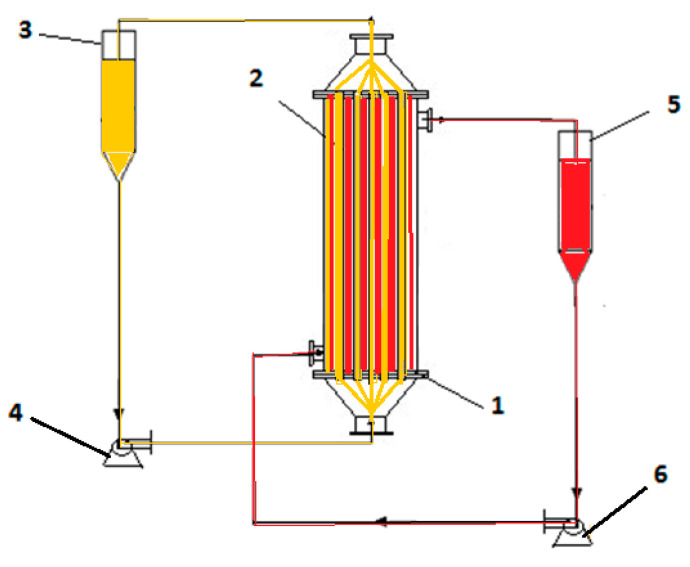
Installation used for the osmium nanoparticles–polymer composite membranes (Os_np_–PM): **1**—permeation module, **2**—polypropylene hollow fiber, **3**—OsO_4_–*t*-butanol solution reservoir, **4**—OsO_4_–*t*-butanol solution pump, **5**—10–undecenoic acid solution reservoir, and **6**—10–undecenoic acid solution.

Osmium tetroxide (OsO_4_) reduction method in a hollow fiber membrane contactor was used to obtain osmium nanoparticle–polymer composite membranes. The reduction reaction takes place in a permeation module with polypropylene hollow fiber membrane (Figure 1) [39,40]. Using the permeation module 1, the OsO_4_ solution in tert-butyl alcohol is introduced through the polypropylene hollow fiber membrane 2, from the reservoir 3 and with the help of the recirculation pump 4—the yellow circuit. The alcoholic solution (*iso*-propyl alcohol, *tert*-butyl alcohol and *n*-decyl alcohol) of 10–undecenoic acid (UDA) from the reservoir 5 is circulated through the recirculation pump 6 so as to connect with the outside of the capillary fibers—the red circuit.

**Figure 2:**
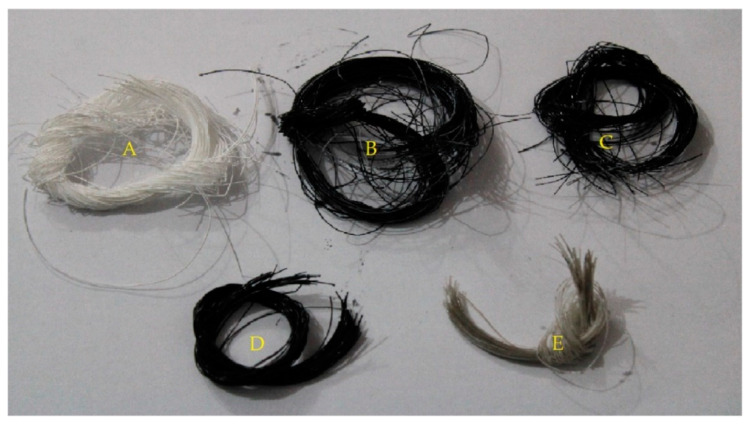
The aspect of the composite nanoparticles–polymer membranes (Os_np_–PM): **A**—polypropylene hollow fiber membranes support (PP); **B**—Os–PPM*i* from OsO_4_–*i*-propanol–10–undecenoic acid; **C**—Os–PPM*t* from OsO_4_–*t*-butanol–10–undecenoic acid; **D**—Os–PPM*n* from OsO_4_–*n*-decanol–10–undecenoic acid; **E**—Os–PPM*p* from OsO_4_–pure 10–undecenoic acid.

The osmium nanoparticles formation reaction, according to Equation (1), leads to the obtaining of composite nanoparticles–polymer membranes (Os_np_–PM) (Figure 2).

The nanocomposite membrane samples containing osmium are characterized morphologically by scanning electron microscopy (SEM) and high-resolution SEM (HR–SEM); structurally by energy-dispersive spectroscopy analysis (EDAX), Fourier transform infrared (FTIR) spectroscopy; and in terms of process performance, by thermal gravimetric analysis by differential scanning calorimetry (TGA, DSC) and in a redox reaction of an organic marker, *p*–nitrophenol (PNP) to *p*–aminophenol (PAP).

#### 2.2.2. The Reduction Process of *p*–Nitrophenol to *p*–Aminophenol on Membrane Contactor

**Figure 3:**
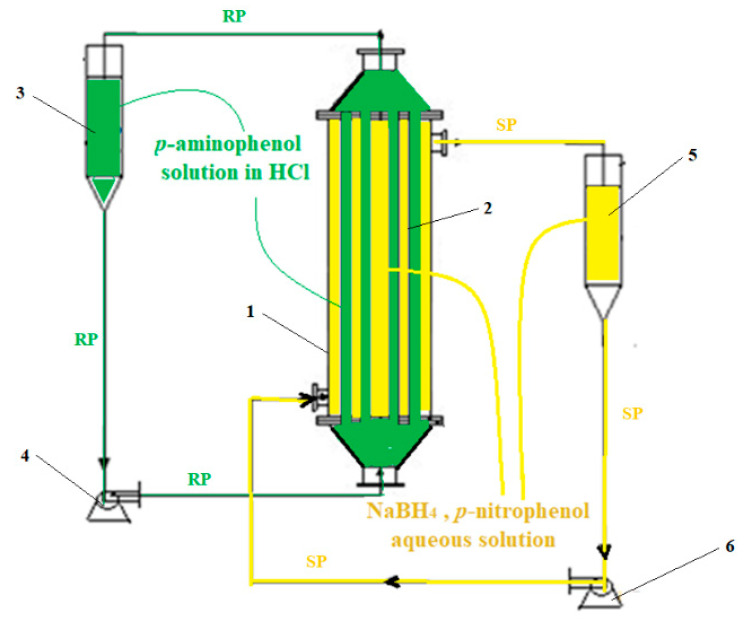
The schematic presentation of the installation: **1**—membrane contactor module; **2**—composite nanoparticles–polymer membranes (Os_np_–PM); **3**—receiving phase (HCl solution); **4**—receiving phase pump; **5**—source phase (aqueous NaBH_4_–*p*–nitrophenol solution); **6**—source phase.

The composite nanoparticles–polymer membranes (Os_np_–PM) constitutes itself as the central element of the contactor-type membrane reactor (Figure 3), which was previously presented in detail [36,37,38]. This contactor type assumes fixing the hollow fiber composite nanoparticles–polymer membranes (Os_np_–PM), in such a way as to allow separate circulation through the outside and inside of the membranes, respectively.

The source solution of *p*-nitrophenol, which also contains sodium borohydride (SP), is circulated in the permeator 1 (Figure 2) through the outside of the membranes, and the hydrochloric acid receiving solution (RP) is recirculated through the composite membranes.

The monitoring of the process is achieved by taking samples of 1 mL of *p*–nitrophenol solutions, which are periodically spectrophotometrically analyzed (CamSpec Spectrophotometer—Spectronic CamSpec Ltd., Leeds, UK) [41,42].

The conversion efficiency (*η*%) of *p*–nitrophenol to *p*–aminophenol is calculated either in terms of concentration (3) or using the absorbance of the samples (4) [43,44,45]:

(3) $$\eta \left(\%\right)=\frac{\left(c_{0}-c_{f}\right)}{c_{0}}\cdot 100$$

*c_f_* being the final concentration of the solute (PNP or PAP), *c*_0_ the initial concentration of solute (PNP or PAP), and:

(4) $$\eta \left(\%\right)=\frac{\left(A_{0}-A_{s}\right)\;}{A_{0}}\;\cdot 100$$

*A*_0_ being the initial sample solution absorbance (PNP or PAP) and *A_s_* the current sample absorbance (PNP or PAP).

### 2.3. Apparatus and Instruments

The preparation of the composite membrane samples for examination by scanning electron microscopy consisted of metallization with a surface layer of gold under vacuum, which was then examined with Hitachi S4500 system (Hitachi High-Technologies Europe GmbH, Krefeld, Germany). For elemental analysis and energy-dispersive spectroscopy analysis (EDAX), using the same apparatus, the samples were not metallized.

Thermal behavior was followed with a STA 449C F3 system, TG-DSC (thermogravimetric–differential scanning calorimetry) from Netzsch (NETZSCH-Gerätebau GmbH, Selb, Germany), between 20 and 900 °C, in dynamic (50 mL/min) air atmosphere. The evolved gases were transferred through heated transfer lines and analyzed on the fly with the help of a FTIR Tensor 27 from Bruker (Bruker Co., Ettlingen, Germany), equipped with an internal thermostatic gas cell.

Spectrophotometric analyses for *p*-nitrophenol (PNP) or *p*-aminophenol (PAP) solutions, as well as for alcoholic solutions of 10–undecenoic acid, were performed using a Spectrometer CamSpec M550 (Spectronic CamSpec Ltd., Leeds, UK). For validation, the measurements were repeated on the Varian Cary 50 (Agilent Technologies Inc., Santa Clara, CA, USA) spectrometer.

pH monitoring was performed with electrodes and devices from Hanna Instruments Ltd., Leighton Buzzard, UK.

Atomic absorption spectrometry, with a dedicated cathode lamp, followed the concentration of osmium in the membrane system using specific apparatus (AAnalyst 400 AA Spectrometer) and software from Perkin Elmer (Perkin Elmer Inc., Waltham, MA, USA).

## 3. Results and Discussions

Obtaining composite membranes of osmium nanoparticles–polymer type has aroused special interest, because they can be used both in reduction and oxidation processes [15,16].

In this paper, a method is considered to obtain a liquid membrane based on aliphatic-alcohol-containing dispersed osmium nanoparticles, on a resistant physical-chemical support consisting of polypropylene hollow fiber membranes [46,47]. Osmium nanoparticles were obtained directly on the membrane support (Figure 2) by reducing osmium tetroxide, from *t*–butanol with 10–undecenoic acid (UDA) to *i*–propanol, *t*–butanol or *n*–decanol solution.

The supported liquid membranes that have been obtained were characterized morphologically and structurally, and for the validation of the process performances, the reduction of an established target substance was chosen [47,48,49,50], *p*–nitrophenol (PNP), to *p*–aminophenol (PAP).

The choice of *p*-nitrophenol (PNP) as target substance relied on technical-economic and environment arguments (high value of PAP reaction product, PNP toxicity, need to remove PNP from effluents), as well as analytical practice arguments concerning accessible and reliable analysis of solutions and the existence of a multitude of comparative experimental data on the reaction process.

### 3.1. Characterization of Obtained Composite Membranes (Os–A–PPM)

**Figure 4:**
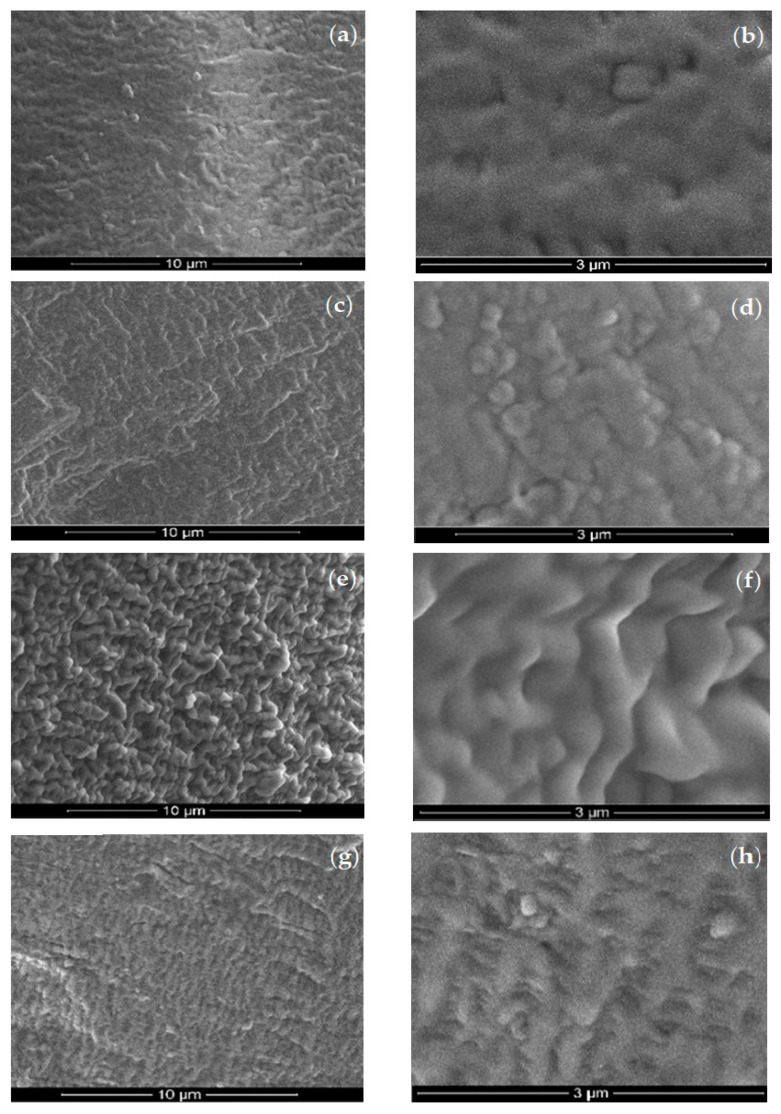
Scanning electron microscopy (SEM) of the obtained composite membranes (Os–PPM): (**a**,**b**) Os–PPM*i* from OsO_4_–*i*-propanol–10–undecenoic acid; (**c**,**d**) Os–PPM*t* from OsO_4_–*t*-butanol–10–undecenoic acid; (**e**,**f**) Os–PPM*n* from OsO_4_–*n*-decanol–10–undecenoic acid; (**g**,**h**) Os–PPM*p* from OsO_4_– pure 10–undecenoic acid. Resolutions: (**a**,**c**,**e**,**g**) ×10,000; (**b**) ×50,000; (**d**) ×40,000; (**f**) ×50,000; (**h**) ×50,000.

The morphological aspects observed, at ×10,000 and ×50,000 magnitude, by scanning electron microscopy (SEM) and high-resolution SEM (HR-SEM) of the composite membranes (Os–A–PPM) are presented in Figure 4. The supported liquid membranes based on *iso*-propyl alcohol, *tert*-butyl alcohol and *n*-decyl alcohol and 10–undecenoic acid (Os–PPM*i*, Os–PPM*t* and Os–PPM*p*) have a discontinuous appearance highlighted in Figure 4a,c,h, at a resolution that shows the entire surface of the membranes (×10,000), more visible in the details with the resolution ×50,000 (Figure 4b,d,h). The *n*–decanol membrane (Os–PPM*n*) has a continuous appearance (Figure 4e,f), no longer observing the pores of the support or aggregates of nanoparticles. The overall images of the membranes (Figure 2) are suggestive, being correlated with the microscopic observations (SEM).

**Scheme 1:**
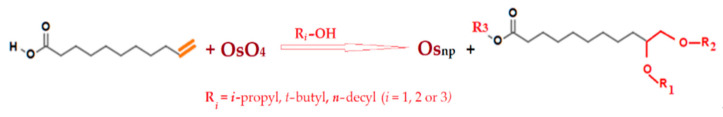
The scheme of reduction of osmium tetroxide with undecenoic acid.

The reduction reaction scheme (Scheme 1) [15] occurs at a significantly higher rate when using *i*–propanol than in the case of *t*–butanol or *n*–decanol. In the case of pure 10–undecenoic acid, the rate of nanoparticle formation is very low, with the process of obtaining membranes lasting over 3 h.

The kinetics of the reduction reaction (5) deserve special attention and may lead to useful observations for the use of membranes obtained in oxidation reactions catalyzed by the osmium nanoparticles thus obtained.

From a qualitative point of view, it can be stated that the dissociation of 10-undecenoic acid is favored by the decrease in the alkyl carbon chain in the structure of the alcohol, which is the reactional medium. The fact that the reduction reaction of osmium tetroxide in pure 10–undecenoic acid is very slow indicates an intramolecular assistance of the carboxyl group in reducing the double bond. This observation is consistent with oxidation studies with osmium tetroxide or osmium catalysts of unsaturated linear ω-acids or their esters when cyclic compounds are obtained [51,52,53,54,55].

**Figure 5:**
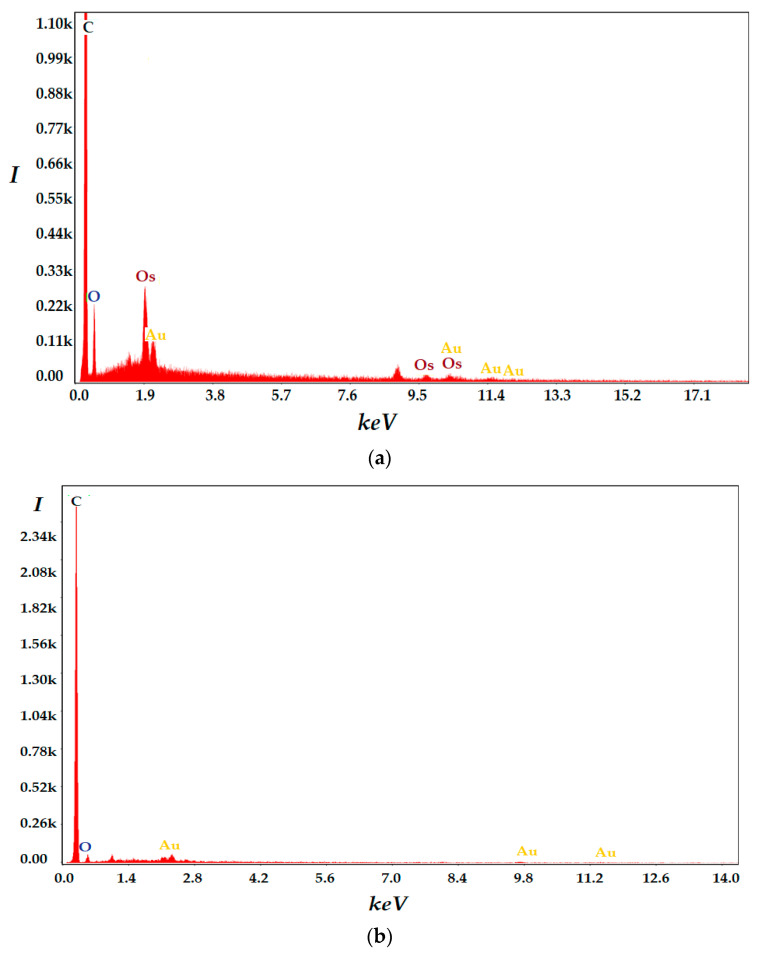
Formal aspect elemental analysis obtained by energy-dispersive spectroscopy analysis (EDAX) on: (**a**) composite membranes, and (**b**) polypropylene hollow fiber membranes support.

The results obtained by elemental analysis (EDAX) are presented both in Figure 5, which illustrates the general appearance of spectra for the obtained membranes and the polymeric support, and in Table 1, which presents the surface analysis for the membranes and the polypropylene hollow fiber membranes support. The content of osmium on the membrane surface ranges from 0.42% to 3.97% gravimetric, being at its maximum for Os–PPM*n* (OsO_4_–*n*-decanol–10–undecenoic acid) and minimum for Os–PPM*p* (OsO_4_–pure 10–undecenoic acid).

These observations are consistent with the rate of nanoparticles formation but also with the stability of the composite membranes obtained. In particular, the membrane based on *n*–decanol is the most stable, a fact directly observed by scanning electron microscopy (Figure 4e,f).

**Figure 6:**
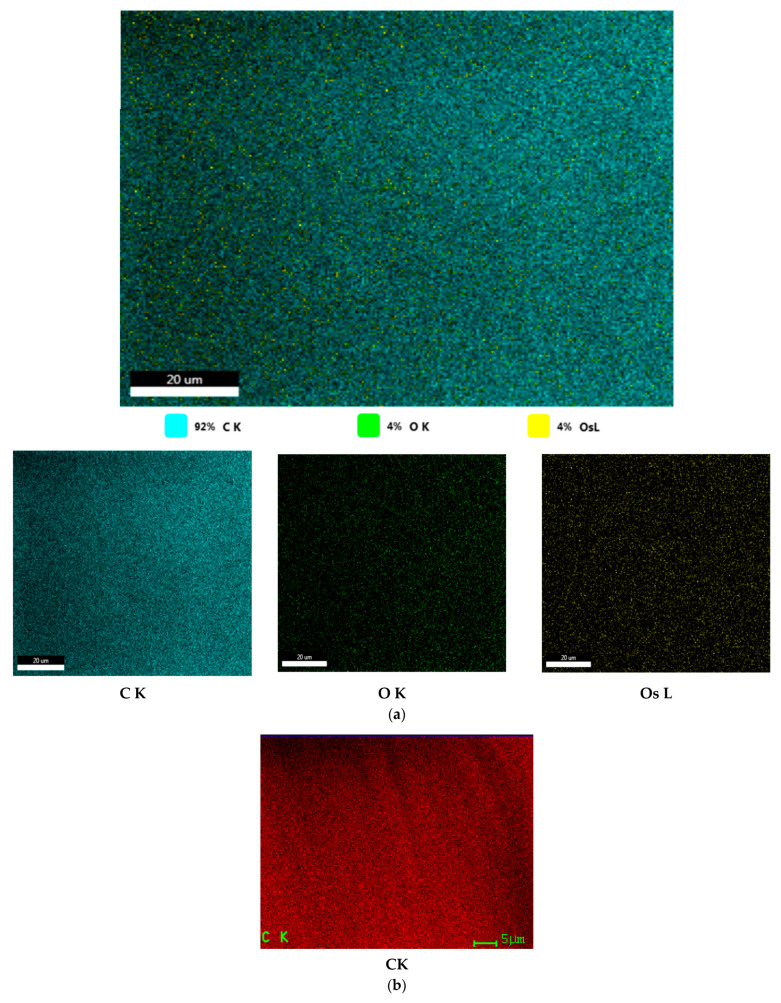
Elements distribution map obtained by energy-dispersive spectroscopy analysis (EDAX) on: (**a**) osmium nanoparticle–polymer composite membranes, obtained in *n*-decanol (Os–PPM*n*); (**b**) polypropylene polymer support.

In order to follow the superficial distribution of the elements on the surfaces of the membranes, the map obtained by energy-dispersive spectroscopy analysis is presented for the most uniform structure, Os–PPM*n* (Figure 6).

The liquid membrane on the support contains mostly carbon (approx. 83–92% by mass) and oxygen (approx. 7.5% and 13% by mass), the uniformly dispersed osmium being a minority (between about 0.4% and 4% by mass) (see Table 1). These values are very close to previously presented data [15,16] that indicate good results, at these concentrations, both in oxidative catalysis and in the reduction of organic compounds. The polymer support has an elemental composition as follows (see Table 1—specific elemental contents, and Figure 6b—carbon map elemental distribution): carbon (cca. 96.94%), hydrogen, and oxygen (cca. 2.9%). The oxygen content is due to conditioning polyol compound, used for maintaining the fibers’ characteristics.

**Figure 7:**
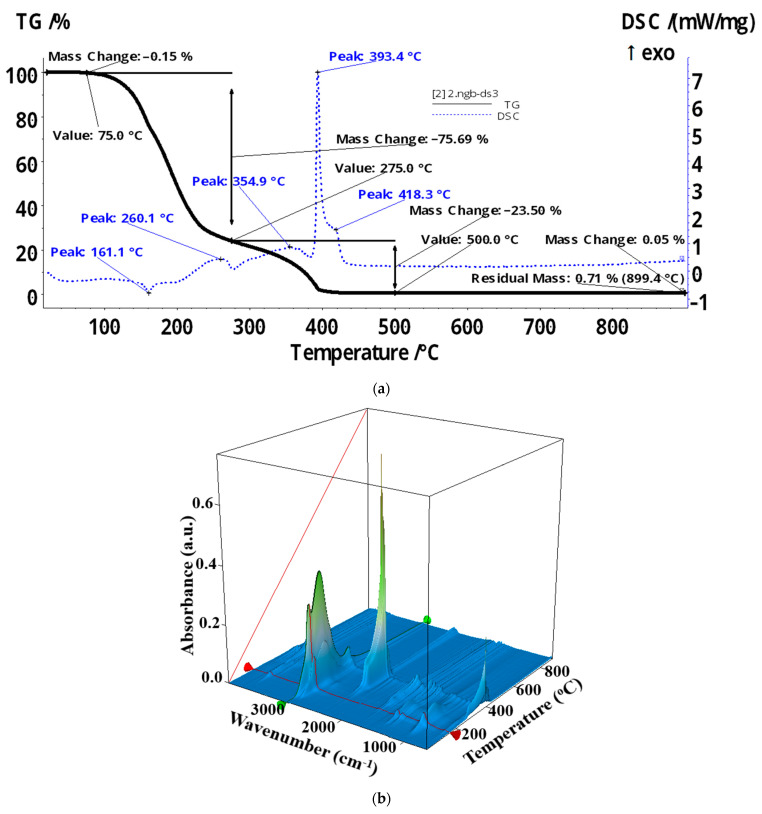
Thermal characteristics (**a**) and the 3D plot of FTIR spectrum vs. temperature (**b**) for the evolved gases from the Os–PPM*i* sample.

**Figure 8:**
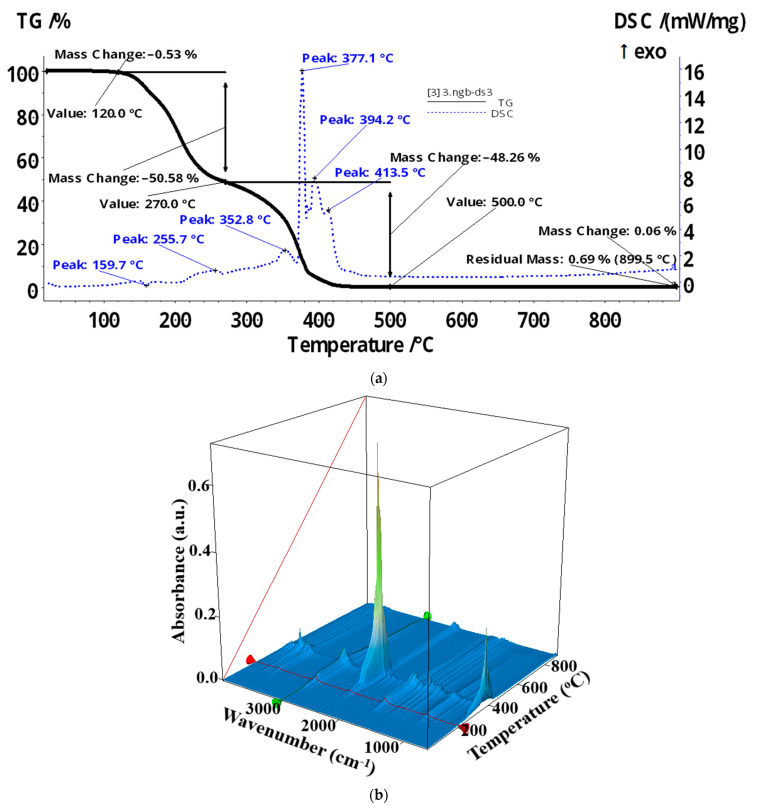
Thermal characteristics (**a**) and the 3D plot of FTIR spectrum vs. temperature (**b**) for the evolved gases from the Os–PPM*t* sample.

**Figure 9:**
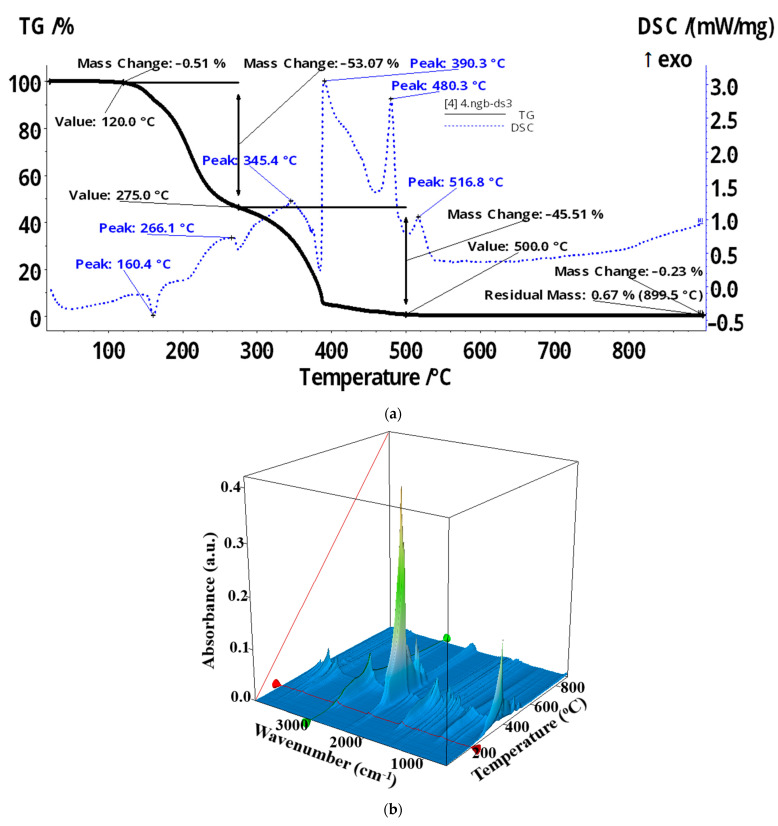
Thermal characteristics (**a**) and the 3D plot of FTIR spectrum vs. temperature (**b**) for the evolved gases from the Os–PPM*n* sample.

**Figure 10:**
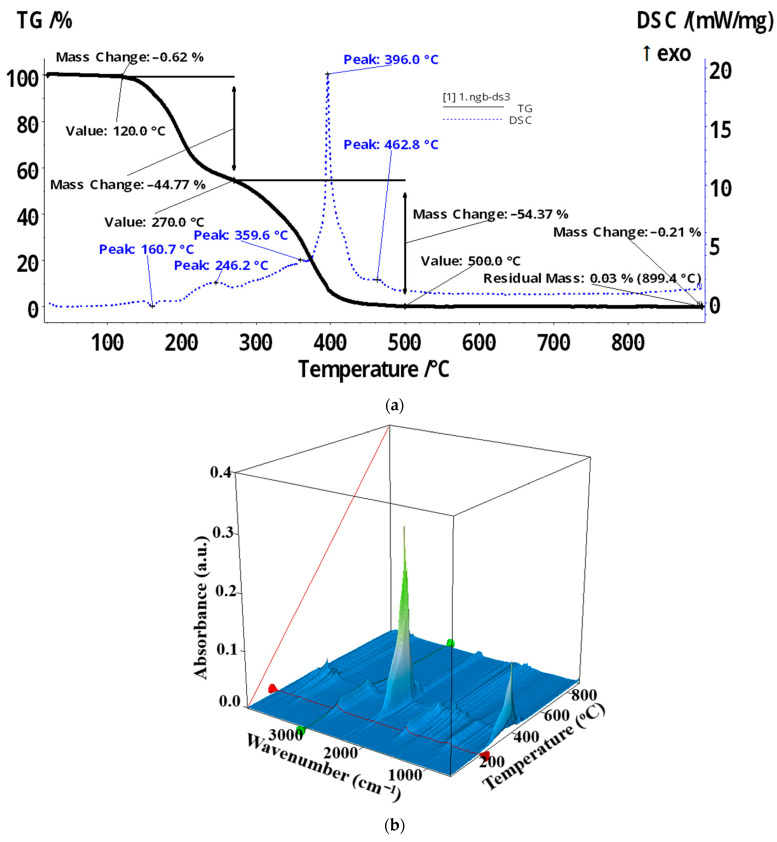
Thermal characteristics (**a**) and the 3D plot of FTIR spectrum vs. temperature (**b**) for the evolved gases from the Os–PPM*p* sample.

The thermal study combined with the infrared spectrometry of the supported liquid membranes (Os–alcohol–PPM) followed both their stability with increasing temperature and the identification of desorption and decomposition products (Figure 7, Figure 8, Figure 9 and Figure 10).

The sample Os–PPM*i* is stable up to 75 °C (Figure 7). From 75–275 °C the solvent is eliminated from the sample, the recorded mass loss being 75.69%. In the same interval, a small endothermic effect can be observed on the DSC curve, with minimum at 161.1 °C, representing the melting of the polypropylene fibers. The small exothermic effect from 260.1 °C is due to the partial degradation by oxidation of some of the organic part.

The oxidative degradation of polypropylene mass occurs mostly after 275 °C, when 23.50% of initial mass is removed. The DSC curve presents multiple peaks, at 354.9, 393.4 and 418.3 °C, all from exothermic effects, the strongest one being attributed to the mass oxidation of the polymer, while the last one is attributed to the burning of carbonaceous mass.

The Os–PPM*t* sample exhibits a good stability up to 120 °C (Figure 8). From 120–270 °C the sample loses 50.58% of its initial mass. In the same temperature interval on the DSC curve, a small endothermic effect can be observed at 159.7 °C, representing the melting of the polymeric fibers. The small exothermic effect from 255.7 °C can represent the partial oxidation of the organic part. The oxidative degradation of polypropylene mass occurs mostly after 270 °C, when 48.26% of initial mass is removed. The DSC curve presents multiple peaks, at 352.8, 377.1, 394.2 and 413.5 °C, all from exothermic effects, the strongest one being attributed to the mass oxidation of the polymer, while the last one is attributed to the burning of carbonaceous mass.

The Os–PPM*n* sample is stable up to 120 °C (Figure 9). From 120–275 °C the recorded mass loss is 53.07%. In the same interval, a small endothermic effect can be observed on the DSC curve, with a minimum at 160.4 °C, representing the melting of the polypropylene fibers. The weak exothermic effect at 266.1 °C is due to the partial oxidation of the organic part. The oxidative degradation of polypropylene mass occurs mostly after 275 °C, when 45.51% of initial mass is removed. The DSC curve presents multiple peaks, at 345.4, 390.3, 480.3 and 516.8 °C, all from exothermic effects, attributed to the mass oxidation of the polymer and to the burning of carbonaceous mass.

The Os–PPM*p* sample is stable in the temperature interval RT–120 °C (Figure 10). From 120–270 °C the sample loses 44.77% of initial mass. On the DSC curve, a weak endothermic effect, at 160.7 °C, can be attributed to the melting of the polypropylene fibers. The small exothermic effect at 246.2 °C can be attributed to the oxidation of the organic part. In between these temperatures, at 188.6 °C, a hardly observable endothermic effect indicates the elimination of undecylenic acid from the sample. The oxidative degradation of polypropylene mass occurs mostly after 270 °C, when 54.37% of initial mass is removed. The DSC curve presents multiple peaks, at 359.6, 396.0 and 462.8 °C, all from exothermic effects, the strongest one being attributed to the mass oxidation of the polymer, while the last one is attributed to the burning of the carbonaceous mass.

All samples present a similar behavior during thermal analysis. The solvent impregnated in the hollow polypropylene fibers is eliminated at low temperatures, starting as low as 75 °C (*i*–propanol), up to 300 °C (for UDA). Osmium nanoparticles are oxidized even at room temperature; therefore, after 130 °C we can consider all traces of OsO_4_ to be eliminated from the samples. The polypropylene fibers will melt around 160 °C and start to decompose after 200 °C, with higher intensity after 250 °C [39]. The carbonaceous mass obtained after the partial degradation of the organics will usually burn around 400–500 °C, mainly eliminating CO_2_, together with small quantities of CO and H_2_O.

**Figure 11:**
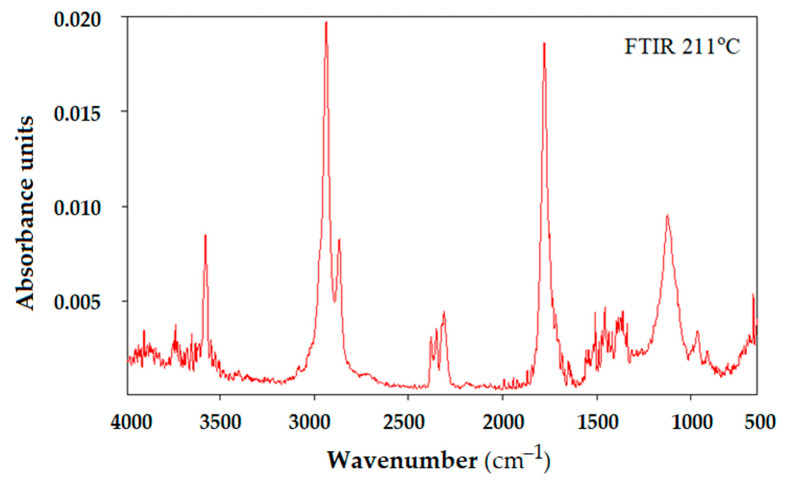
The identification of 10–undecenoic acid (UDA) in samples, at 211 °C.

The FTIR spectra (Figure 7b, Figure 8b, Figure 9b and Figure 10b) allowed the identification of the alcohols in which the oxidation reaction took place, but especially of 10–undecenoic acid through its characteristic functions (Figure 11), left in excess after the reduction of osmium tetroxide. The strip specific to the hydroxyl groups (–O–H) is narrow (3500–3600 cm^−1^), due to the temperature at which the determination was performed (211 °C), when the hydrogen bonds no longer manifest. All other specific functions (C–H, C=O, C–O, –C=C–) appear at the wavenumber values specific to 10–undecenoic acid.

### 3.2. Performances of the Composite Membranes (Os–A–PPM) in the Process of Reducing P-Nitrophenol

Nitrophenols are raw materials in products such as pharmaceuticals, pesticides, colorants, herbicides, paints and pigments, leather and wood, and their presence, even in traces, in aqueous effluents is undesirable and strictly regulated [56,57]. In particular, in addition to its recognized toxicity, *p*-nitrophenol (PNP) also has carcinogen potential, which has led to extensive studies of its removal from aqueous solutions [58,59]. For this study, hydrogenation catalyzed by osmium nanoparticles was chosen, along with recuperative separation, to form *p*-aminophenol (PAP), which constitutes a valuable raw material for the manufacture of pharmaceuticals, having multiple other applications [60]. Previously [15,16], the heterogeneous catalytic reduction with hydrogen gas was presented, justified by the lack of conventional impurities of hydrogenation processes and Fe, Ni, Sn or Cu ions. In this work, the choice was made to reduce *p*-nitrophenol to *p*-aminophenol with sodium borohydride in aqueous solution. It has been considered an environmentally friendly and clean manufacturing process for the removal of *p*–nitrophenol and the obtaining of *p*–aminophenol [61,62,63,64,65,66].

**Figure 12:**
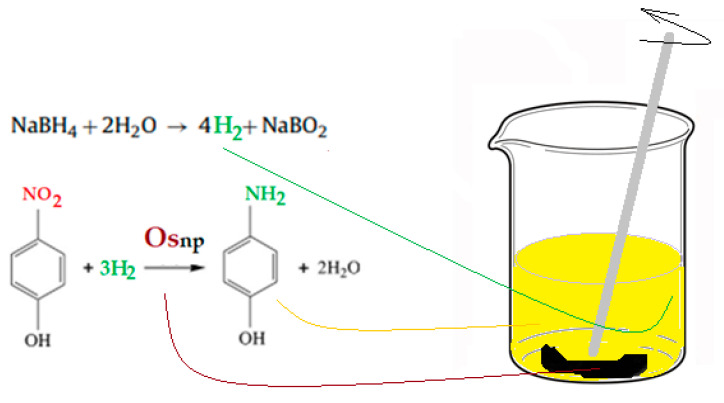
Schematic representation of the reduction processes, with Os–PPM composite membranes.

The performances of the obtained membranes were tested during the catalytic hydrogenation of *p*–nitrophenol (PNP) to *p*–aminophenol (PAP). It was conducted in a 500 mL cylindrical vessel using 300 mL aqueous solution of PNP (2 g/L), mechanically stirred, in which 1 g of Os–PPM membrane was introduced, under a slight stirring using a glass rod. Under continuous slow stirring, a freshly prepared NaBH_4_ solution (10 g/L) was added at 25 °C. The reaction, according to the scheme presented in Figure 12, was followed spectrophotometrically, and the obtained results allowed the determination of the reaction rate constant, which is close to those known for catalytic materials (Table 2). The slightly lower performances are dependent on the surface concentration of the osmium nanoparticles (Table 1), but especially on the nature of the organic coating of the membranes.

**Figure 13:**
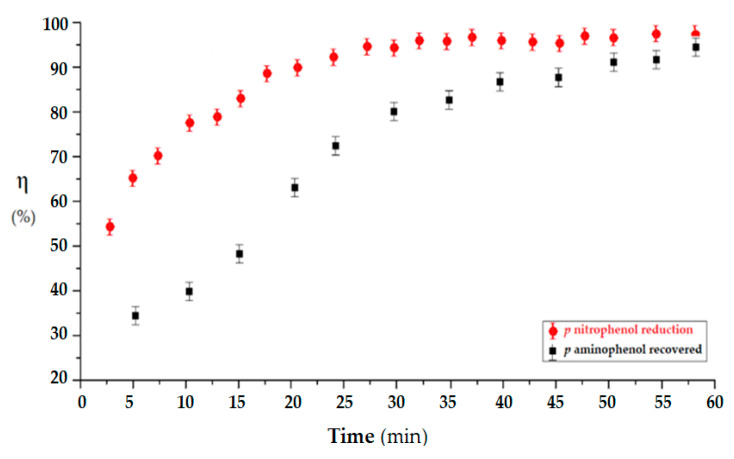
The evolution of the transformation efficiency of *p*–nitrophenol to *p*–aminophenol (in red) and of the recovery efficiency of *p*–aminophenol (in black).

Given the need for the recuperative separation of *p*-aminophenol from the reaction mass, the decisive step for the speed of the entire process is the diffusion through the osmium nanoparticles–alcohol–polymer composite membrane (Os–PPM), from the source phase to the receiving phase (Figure 12 and Figure 13). Thus, it is observed that the transformation of PNP is fast, being practically complete after 25 min, while the PAP recovery proceeds much slower (Figure 12). At the end of the PNP reduction, the recovery efficiency reaches 70%, with its transport to the receiving phase starting to increase, so that after about 60 min it reaches about 96%. Figure 13 illustrates the results obtained for the transformation efficiency of *p*-nitrophenol to *p*-aminophenol and for the recovery efficiency of *p*-aminophenol. The recovery efficiency of the *p*-aminophenol depends primarily on the type of alcohol from which the membrane was formed.

Over the entire time interval, the recovery efficiencies respect the following order:

*η*_Os–PPM*i*_ > *η*_Os–PPM*t*_ > *η*_Os–PPM*m*_

**Figure 14:**
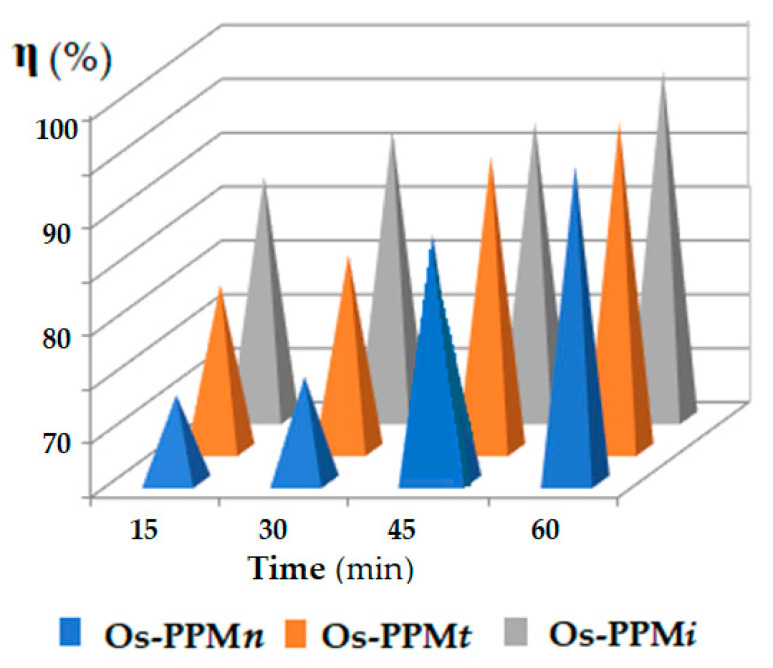
Evolution of *p*–aminophenol recovery efficiency in the case of osmium nanoparticles–alcohol–polymer composite membranes (Os–PPM).

However, at the end of the studied time interval, the recovery efficiencies are almost identical, at approx. 90% (Figure 14).

**Figure 15:**
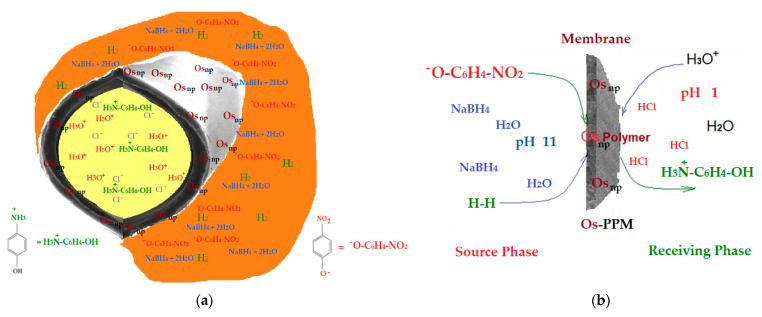
Formal presentation (**a**) and the mechanism of catalytic reduction of *p*–nitrophenol (**b**).

The obtained results allow for elaborating a formalism of reaction and mass transfer through the membranes made of osmium nanoparticles–*n*-decanol–polymer support (Os–PPM*n*) (Figure 15a).

The phenolate (pH = 11) reduction process is developed in the source phase, at the interface with the membrane containing the osmium nanoparticles, with the hydrogen formed in situ by the reaction of sodium tetra-hydroborate with water. The reaction product, *p*–aminophenolate anion, traverses the membrane interface to the receiving phase, where it is solubilized by the formation of the hydroxyl–phenyl–ammonium cation (Figure 15b). The pH gradient ensures the immobilization of the reaction product in the receiving phase.

## 4. Conclusions

The subject of the present study is the realization of a composite membrane in which the polymeric matrix is the polypropylene hollow fiber, and the active component is the osmium nanoparticles obtained by reducing an alcoholic solution of osmium tetroxides directly on the polymeric support. The method of reducing osmium tetroxide on the polymeric support is based on the use of 10–undecenoic acid (10–undecylenic acid) (UDA) as a reducing agent.

Four types of osmium nanoparticles–alcohol–polymer composite membranes (Os–PPM) were prepared: Os–PPM*i* from OsO_4_–*i*-propanol–10–undecenoic acid, Os–PPM*t* from OsO_4_–*t*-butanol–10–undecenoic acid, Os–PPM*n* from OsO_4_–*n*-decanol–10–undecenoic acid and Os–PPM*p* from OsO_4_–pure 10–undecenoic acid, which by testing in the catalytic reduction reaction with sodium tetraborate solution of *p*-nitrophenol to *p*-aminophenol led to constant catalytic rates between 2.04 × 10^−4^ mmol s^−1^ and 8.05 × 10^−4^ mmol s^−1^.

The polymer composite membranes containing osmium nanoparticles (Os–NP) were characterized morphologically, using scanning electron microscopy (SEM), high-resolution SEM (HR–SEM), and structurally, using energy-dispersive spectroscopy analysis (EDAX) and Fourier transform infrared (FTIR) spectroscopy.

The best performance is obtained for osmium nanoparticles–*n*-decanol–polypropylene hollow fiber membranes (Os–PPM*n*).

## Figures and Tables

**Table 1:** Surface analysis for the obtained osmium nanoparticle–polymer composite membranes.

Membrane	Element	Weight %	Atomic %	Net Int.	Error %	K Ratio	Z	A	F
Os–PPM*i*	C K	89.64	93.09	946.60	5.45	0.7571	1.0075	0.8383	1.0000
	O K	8.72	6.80	27.90	52.16	0.0074	0.9696	0.0873	1.0000
	OsL	1.64	0.11	25.70	60.43	0.0120	0.5837	1.2096	1.0361
Os–PPM*t*	C K	84.89	89.65	788.70	8.56	0.6724	1.0107	0.7837	1.0000
	O K	12.86	10.20	40.70	54.86	0.0115	0.9729	0.0918	1.0000
	OsL	2.25	0.15	34.30	65.33	0.0165	0.5862	1.2157	1.0306
Os–PPM*n*	C K	83.97	90.02	811.30	5.28	0.6410	1.0152	0.7519	1.0000
	O K	12.06	9.71	41.10	31.83	0.0108	0.9778	0.0913	1.0000
	OsL	3.97	0.27	61.40	44.29	0.0287	0.5905	1.2014	1.0214
Os–PPM*p*	C K	92.10	94.22	2502.90	1.85	0.8207	1.0038	0.8877	1.0000
	O K	7.49	5.75	57.20	12.51	0.0062	0.9656	0.0859	1.0000
	OsL	0.42	0.03	16.10	20.37	0.0031	0.5802	1.2122	1.0550
PPM support	C K	94.79	96.94	3.09	652.16	0.8250	1.0027	0.8679	1.000
	O K	3.78	2.90	18.00	25.03	0.0042	0.9887	0.1117	1.000
	OsL	-	-	-	-	-	-	-	-

**Table 2:** Comparative data on the ‘catalytic rate constant (*k*)’ for the catalytic reduction processes.

Catalytic Material	*k* (mmol·s^−1^)	Reference
Os–PPM*i*	2.04 × 10^−4^	This study
Os–PPM*t*	2.89 × 10^−4^	
Os–PPM*n*	8.05 × 10^−4^	
Os–PPM*p*	1.01 × 10^−4^	
Nanofibers PtNi/SiO_2_	434 × 10^−3^	[63]
Nanofibers Ni/SiO_2_	18 × 10^−3^	
Nanofibers Pt/SiO_2_	55 × 10^−3^	
Ni–Ca–Al_2_O_3_	2.85 × 10^−3^	[64]
Ni catalysts	1.02 × 10^−3^	
Ni–Al_2_O_3_	1.42 × 10^−3^	
Nanofibers Ni–P 0.25/NFM 4.55	18.04 × 10^−3^–26.84 × 10^−3^	[65]

## Data Availability

Data is contained within the article.
